# Increasing Patient Engagement in Rehabilitation Exercises Using Computer-Based Citizen Science

**DOI:** 10.1371/journal.pone.0117013

**Published:** 2015-03-20

**Authors:** Jeffrey Laut, Francesco Cappa, Oded Nov, Maurizio Porfiri

**Affiliations:** 1 Department of Mechanical and Aerospace Engineering, New York University Polytechnic School of Engineering, Six MetroTech Center, Brooklyn, NY, United States of America; 2 Department of Mechanical and Aerospace Engineering, Sapienza University of Rome, Via Eudossiana 18, Rome 00185, Italy; 3 Department of Business and Management, LUISS Guido Carli University, Viale Pola 12, Rome 00198, Italy; 4 Department of Technology Management and Innovation, New York University Polytechnic School of Engineering, Five MetroTech Center, Brooklyn, NY, United States of America; Cornell University, UNITED STATES

## Abstract

Patient motivation is an important factor to consider when developing rehabilitation programs. Here, we explore the effectiveness of active participation in web-based citizen science activities as a means of increasing participant engagement in rehabilitation exercises, through the use of a low-cost haptic joystick interfaced with a laptop computer. Using the joystick, patients navigate a virtual environment representing the site of a citizen science project situated in a polluted canal. Participants are tasked with following a path on a laptop screen representing the canal. The experiment consists of two conditions: in one condition, a citizen science component where participants classify images from the canal is included; and in the other, the citizen science component is absent. Both conditions are tested on a group of young patients undergoing rehabilitation treatments and a group of healthy subjects. A survey administered at the end of both tasks reveals that participants prefer performing the scientific task, and are more likely to choose to repeat it, even at the cost of increasing the time of their rehabilitation exercise. Furthermore, performance indices based on data collected from the joystick indicate significant differences in the trajectories created by patients and healthy subjects, suggesting that the low-cost device can be used in a rehabilitation setting for gauging patient recovery.

## Introduction

Rehabilitation is the structured process undertaken toward adapting to unforeseen changes resulting from trauma or disease [1]. For individuals with severe motor dysfunction, it is a necessary step to recover self-reliance [2]. In this context, repetitive exercises have been shown to offer effective rehabilitation treatments in stroke patients [3–7], but often at the cost of a large time-commitment from both the physical therapist and patient.

Robotic devices have been identified as an effective means to administer [8–10] or complement these therapeutic treatments [11], and neuroprosthetics [12, 13] may directly enhance motor function. By shifting to robotics-based treatments, physical therapists may be able to simultaneously treat a greater number of patients and obtain performance measures from the data gathered through the robotic device’s sensors [14], which has been demonstrated to be useful in assessing motor performance [15, 16]. Within these treatments, measurement of a patient’s movement is typically of interest, as it allows for gauging recovery through the comparison of these pathological movements with normal movements [17]. For instance, sensors on robotic devices can collect the duration of completing a task, the smoothness of patients’ movements, and the jerkiness of the resulting trajectories, all of which have been shown to be valid indicators of recovery [18, 19] that are often useful to physical therapists for assessing patients’ progress [20].

Although robotic devices have been successfully implemented in therapeutic settings, the patient must ultimately be willing to conform to the prescribed medical advice for the treatment to be effective [21]. Indeed, a central issue in the effectiveness of rehabilitation is patient adherence to prescribed regimens, which is often associated with both improved outcomes of the treatment [22] in addition to increased patient’s satisfaction [23]. In a physical therapy setting, patients tend to be more compliant in performing exercises when they are with a physical therapist than when they are alone [24]. Lack of motivation has been identified as one of the factors causing this noncompliance in physical therapy [25–27] and physical activity [28]. Lack of motivation has also been attributed to non-compliance in elderly patients to perform exercises after discharge from the hospital [29]. Combining motivational enhancement treatment with conventional physical therapy has been shown to bring about increases in compliance with exercises [30]. Resting on these findings, we posit that including additional motivational elements can be a feasible means to increase compliance in rehabilitation regimens.

Many avenues have been investigated for enhancing motivation levels in rehabilitation settings. Simply providing patients with a sensor-based rehabilitation system may intrinsically motivate patients to perform exercises [31]. The use of computer-based activities has further been identified as a promising means to administer therapeutic exercises for children with sensorimotor disorders [32]. Toward increasing the accessibility of robotics-based devices in home settings, the use of an off-the-shelf gaming system has been proposed in [33], whereby the participant interacts with a virtual environment and experiences haptic feedback through the system’s controller. Beyond reducing the cost of rehabilitation, off-the-shelf gaming systems have the potential of enhancing patient engagement and motivation levels in performing exercises [34]. However, as most commercially available video games are designed for entertainment purposes, they may be too fast-paced for the sensorimotor-impaired in a rehabilitation setting [35]. Serious and social games, not aimed primarily at entertainment [36, 37], are more likely to be effective as they can be specifically tailored for rehabilitation settings [38].

An approach alternative to gaming, based on science learning, has been proposed in [39]. Therein, participants navigate a virtual representation of a zoo using a haptic joystick where the presentation of relevant scientific content and force feedback schemes are varied in four different experiments. The system capitalizes on participants’ interest in science, and results show that higher levels of satisfaction are attained when performing exercises that contained the presentation of scientific content. Extending the findings of [39], here we seek to assess if patient motivation to perform rehabilitation exercises is strengthened by shifting from passive scientific learning to active performance of scientific tasks in a citizen science project.

Thousands of non-scientists are actively volunteering time and effort towards scientific tasks in the context of citizen science. In these types of projects, members of the public make contributions to scientific research projects typically by collecting or processing data [40, 41], but may also take on collaborative roles with the scientists leading the project [42]. The task difficulty in citizen science varies in the amount of effort required from the participants. In “Galaxy Zoo,” for example, citizen scientists view images of galaxies collected by astronomers, and classify the type of galaxy in the photo [43].

The idea of enhancing motivation by substituting science learning with scientific tasks stems from the motivational factors associated with each of these activities. Indeed, while motivation for learning is mainly intrinsic (i.e., users exhibit a personal interest) [44], citizen scientists are also motivated by collective factors (i.e., users are motivated by advancing science) [45]. The users of Galaxy Zoo, for example, have been found to be motivated by both their individual interest in the subject of Astronomy, as well as the excitement that they experience in contributing to the scientific project [46]. Based on the effectiveness of including science learning in exercises demonstrated in [39], citizen science is expected to also be a feasible means to enhance participant motivation in rehabilitation.

Herein, we present the development of a robotics-based system that allows for assessing the efficacy of scientific tasks for enhancing participant motivation and willingness to repeat therapeutic exercises. The hardware components of [39], namely a low-cost haptic joystick and a laptop computer, are repurposed and elements of an existing citizen science project developed by our group, “Brooklyn Atlantis” [47], are included for participants to contribute to a research project. Brooklyn Atlantis (www.BrooklynAtlantis.org) is an environmental monitoring project that is focused on the Gowanus Canal in Brooklyn, NY. Data from the canal are captured by an aquatic mobile robot equipped with water quality sensors and cameras and uploaded to a website. Citizen scientists can contribute through the website by analyzing the image data and adding tags, which describe anything that the participant may deem noteworthy in the scene, for example, an animal or a piece of debris.

Through the use of the robotics-based setup coupled with an interactive environment for scientific contribution, the hypothesis that citizen science can provide a motivational incentive for performing rehabilitation exercises is tested. Using an off-the-shelf haptic joystick (Novint Falcon), participants maneuver a cursor about a two-dimensional virtual representation of the Gowanus Canal in two distinct tasks—one with a scientific task, and one without. Furthermore, we investigate the efficacy of the setup to produce data useful for assessing motor performance in the context of rehabilitation.

## Materials and Methods

### Experimental setup

**Fig 1:**
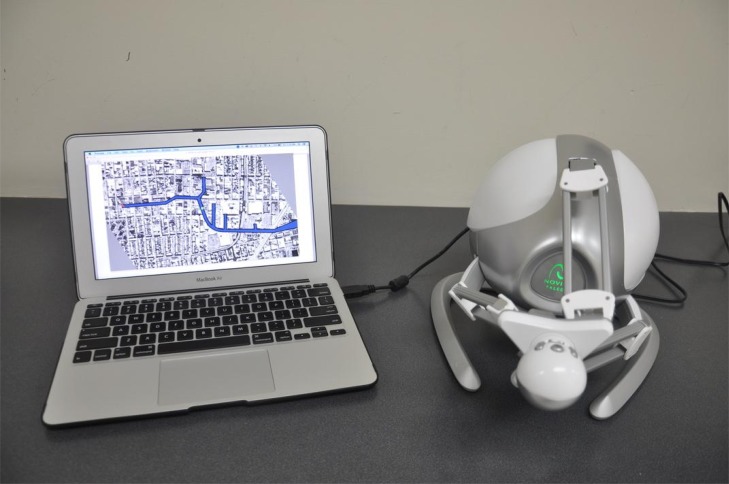
The experimental setup, consisting of a laptop computer and a Novint Falcon haptic device.

The system consists of a laptop computer and the Novint Falcon device (see Fig. 1), making it both low-cost and easily transportable. The Novint Falcon is a delta robot [48], meaning that the end-effector (manipulandum) is unable to rotate, but may translate in all three dimensions. While the Cartesian *x*, *y*, and *z* positions are controlled by moving the manipulandum, four buttons located where the hand grasps the joystick provide additional input from the user. The available workspace provided by the Novint Falcon is similar to those offered by devices used in robot-mediated therapy focused on the elbow and wrist [49, 50]. The laptop computer receives position data from the haptic device and sends force commands to deliver an interactive experience to the user. The system in [39] utilizes Matlab for the program execution, however, the software for this system is written in C++ using the open-source library libnifalcon, offering benefits of lower overall system cost, as well as a significantly faster sampling rate of 1000 Hz.

### Experimental conditions

This study is comprised of two experimental conditions, specifically, task T and task NT. In task T, a set of images captured by the aquatic robot as part of the Brooklyn Atlantis project are presented to each participant at predetermined locations along the canal along with a pop-up keyboard. The participants select a region of interest within each image, and classify, or tag, what they see. In task NT, the participants navigate about the same virtual environment with no images presented.

**Fig 2:**
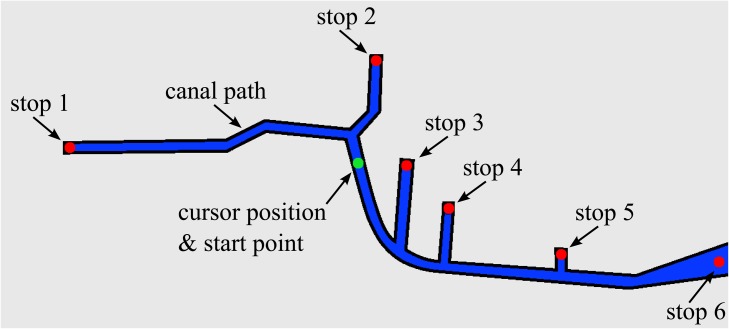
Path displayed to users in map mode, with overlaid dots displaying waypoints (red) and cursor start position (green). The image presented to the participants was superimposed on an aerial photo of the Gowanus Canal.

Upon program execution, the task to be performed is specified as either task T or task NT. Task T presents the participant with a virtual representation of the Gowanus Canal, where the water is highlighted in blue as a path to be followed using joystick input (see Fig. 2). Users are instructed to sequentially hit a series of six waypoints, shown as red dots. Only a single waypoint is displayed at a time in the predetermined order of left to right, thus demanding that each participant follows a similar path. The participant’s current position is displayed by a dot that changes color from green to red as the deviation from the path increases.

While in such “map mode,” the motion of the manipulandum is restricted to a plane, so that the left-right motion of the joystick corresponds to a change in the *x* position of the cursor (along the width of the image), and up-down motion corresponds to change in the *y* position of the cursor (along the height of the image). The force-feedback capabilities of the joystick are utilized to provide another level of interactiveness, whereby a total force consisting of a damping term and a nominal force based on the participant’s distance from the path is given in map mode. More specifically, the nominal force administered is diverging [39], meaning that as the participant’s deviation from the path increases, it becomes more difficult to return the cursor to the path. Indeed, while both assistive and error-amplifying feedback strategies are encountered in rehabilitation, regimens that administer error-amplifying strategies may be more effective [51].

The nominal force for each point on the map is calculated offline and stored as a look-up table to save computation time during program execution. As some sharp changes in the force arise due to sharp corners in the canal, the look-up table is smoothened with a Gaussian filter to deliver a smooth and continuous feeling to the participant. When the trial begins, the cursor is centered on the map, and the participant must move the cursor along the map to the first waypoint, located on the left-most part of the path.

**Fig 3:**
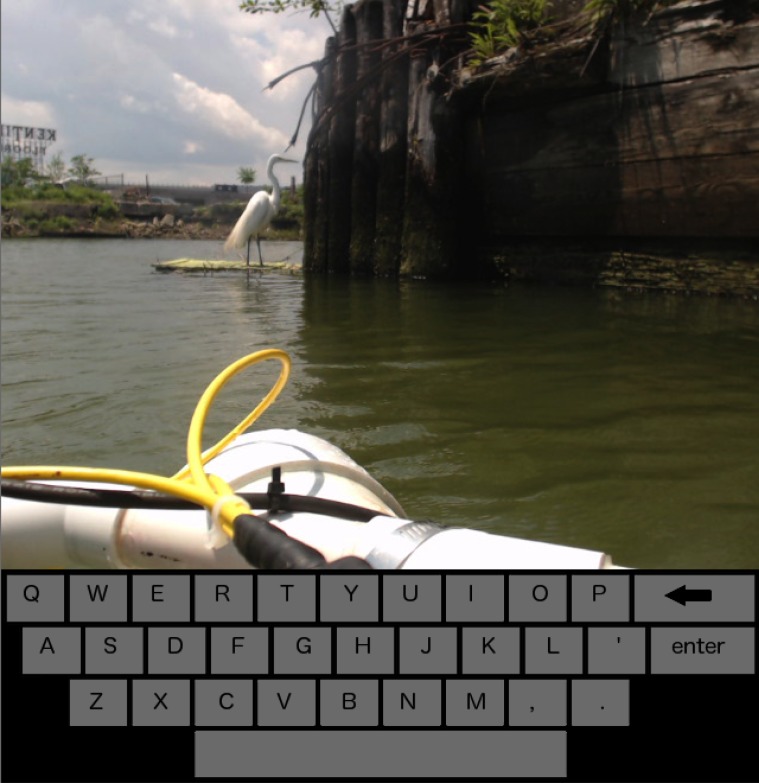
Example of image displayed during tagging mode with on-screen keyboard.

When a waypoint is reached in task T, one of the images captured by the Brooklyn Atlantis robot in the Gowanus Canal is presented with a virtual keyboard in the so-called “tagging mode,” see Fig. 3. The manipulandum of the Novint Falcon in this mode controls the location and size of a box that the participant must position in order to tag an item of interest within the image. Moving the manipulandum left, right, up, and down controls the Cartesian *x* and *y* location of the box within the image, while pushing the manipulandum in or pulling it out controls the size of the box. Clicking the center button sets the position and size of the box in the image, and allows the user to type what the box contains on the virtual keyboard. A red cursor indicates the position of the manipulandum, and a letter is selected by hovering over it and clicking the center button. Once the “enter” key is selected, the image tag is recorded and the user is returned to the route map. To avoid any discontinuities due to the location of the cursor upon exiting tagging mode, the cursor and joystick are automatically repositioned to the current waypoint on the map. Thus, the so-called “waiting mode” is entered, where a wait screen is displayed and the joystick is locked in place for 2 seconds.

For task NT, a trial begins also in map mode and users navigate from the center of the map to each waypoint in sequence. However, unlike task T, reaching the waypoint in task NT simply prohibits the joystick motion for 2 seconds during waiting mode without presenting an image to be tagged.

In both tasks, before continuing to the next waypoint, the participant must “escape” from the current waypoint by exerting a force on the manipulandum greater than a predefined threshold. This is a low valued threshold that simply serves to allow for identifying a deliberate re-entry into route traversal, thereby allowing a direct and sound comparison with the path following performance in both task T and NT. After reaching all six waypoints, and, in the case of task T, tagging all images, the task is complete.

### Post-experiment survey

Following each participant’s completion of both tasks, a nine-question survey is administered. The survey is designed to assess the participant’s preference between the tasks and their perceived difficulty. The first two questions gather basic information about the participant, where question 1 (Q1) asks about the subject’s age and gender, and Q2 asks about the arm used for the exercise. Questions 3–5 ask, respectively, “which task did you like more?,” “which task was more difficult?,” and “if you had to repeat one of the tasks, which would you choose?,” users can respond with either an answer of task T or task NT. Q6 and Q7 are answered by choosing a smiling face, neutral face, or sad face, for the prompts “circle the letter under the face that describes how identifying objects and animals made you feel” and “circle the letter under the face that describes how moving the cursor without identifying animals and objects made you feel.” The response choices to Q8 and Q9 are green, yellow, and red rectangle with “easy,” “medium,” and “difficult” respectively written under each rectangle, and the questions ask the user to rank how easy or difficult identifying objects and animals is and to rank how easy only moving the cursor without identifying objects and animals is.

### Ethics statement

The ethics and medical board of the Children’s Hospital “Bambino Gesù,” (Rome, Italy) approves the experimental protocol and consent procedure. Each participant and one or both of their parents are explained the details of the experiment, and oral informed consent is obtained prior to the experiment execution, while written consent from parents is obtained by hospital staff and securely stored at the Children’s Hospital “Bambino Gesù.”

### Participants

To demonstrate the capability of the proposed setup to deliver meaningful performance indices for rehabilitation purposes, the experiment is carried out on two groups of individuals, that is, a group of 30 patients (age 12.1 ± 4.2 years) enrolled in physical rehabilitation regimens primarily for the upper limb, and a group of 30 healthy individuals (age 10.3 ± 2.8 years).

The inclusion criteria for all participants is a lack of prior experience with the Novint Falcon haptic device or the Brooklyn Atlantis project. In addition, patients are selected by a physical therapist based on their age and the severity of their physical impairments, which are primarily a result of stroke or cerebral palsy. The experimental setup requires a high degree of fine motor skills, as participants must maneuver about a path within a small workspace and be able to select individual letters for tagging purposes. Therefore, patients use whichever arm is more capable of completing the exercises. All of the trials for both patients and healthy subjects are performed at the Children’s Hospital “Bambino Gesù,” in Rome, Italy.

### Experimental protocol

Participants from both the group of healthy subjects and the group of patients are subject to the same protocol. The experimental platform is placed on a desk, and the participant is asked to sit comfortably on a chair in front of the desk. Depending on the hand being used, the Novint Falcon is placed either on the right or on the left of the laptop computer. Prior to beginning the experiment, the participant is given an overview of the Brooklyn Atlantis citizen science project through a video. The order of execution of tasks T and NT are alternated such that an equal number of participants perform task T first as those who perform task NT first within each group.

### Data treatment

As each task is being executed, all data pertinent to the user’s input and state of the program, such as cursor position, force feedback commanded, elapsed time, and operating mode of the program are logged to a datafile. This comprehensive collection of data allows for the assessment of each participant’s performance.

All of the post-processing of the logged data is performed in Matlab, where a script imports the datafile and extracts the relevant data. To filter the position data, a sixth-order butterworth filter with an 11 Hz cutoff frequency is implemented following [52]. The data are partitioned based on the program operating mode (e.g., map mode, waiting mode, or tagging mode), and for both tasks, only the data corresponding to map mode is investigated. Furthermore, since the escape motion of leaving a waypoint often induces a large acceleration, a half-second of data following the escape from each waypoint is also excluded. Partitioning the data in this manner that only includes path following allows for accurate performance comparisons both between groups and between tasks.

### Performance indices

Through the use of the high-resolution sensor data provided in robot mediated therapy, a set of performance indices based on the trajectory created by a participant may be used to quickly quantify their performance. The following metrics, which have been previously used and validated in upper-limb robot-mediated therapy [18, 52, 53], are scored: (i) the normalized path length, which is the length of the path taken by the user divided by the length of the path to be followed [53]; (ii) the mean of the jerk, found by averaging the jerk exhibited at velocities above a minimum velocity threshold *v*_*thresh*_ [52]; (iii) the speed metric, measured as the mean velocity above *v*_*thresh*_ divided by the peak velocity [18]; and (iv) the mean arrest period ratio, which is the proportion of time spent moving at a velocity above *v*_*thresh*_ [18]. In addition to these metrics, the total time spent moving on the path, or path duration, and the total time spent on the task, or task duration, are scored.

### Statistical analysis

For all questions, a Mann-Whitney U-test is used to assess differences between the responses of the two groups, consisting of 30 patients and 30 healthy subjects [54]. To study within group preference, the neutral response is omitted from the analysis of questions Q6–Q9, and for all questions we test the hypothesis that positive and negative responses are equally likely using a chi-square test [55].

For survey questions Q3–Q5, a “positive” response is one that coincides with expectations, while a negative response does not. Specifically, for Q3, which asks which task is preferred, the choice of task T is considered positive. For Q4, which asks to identify the more difficult task, task T is also considered positive, and for Q5, which asks to identify which task the participant would like to repeat, task T is again taken as positive. For Q6 and Q7, the answer choices are a smiling face (positive response), neutral face (neutral response), or sad face (negative response), and for Q8 and Q9, the answer choices are “easy” (positive), “medium” (neutral), and “hard” (negative).

Considering the performance metrics, differences between the two groups are assessed using the non-parametric variant of ANOVA, the Kruskal-Wallis test [54], while within groups and between tasks, the Wilcoxon signed rank test is used [54].

All statistical analyses are performed in Matlab with the significance level set at *p* < 0.05.

## Results

### Delivery of scientific tasks influences individual preference (Fig. 4) Q3–Q5

The results support the hypothesis that individuals prefer the scientific task. When asked to indicate their preferred task (Q3), the responses between the groups are not significantly different (*p* = 0.5517), and both healthy subjects and patients indicate a robust preference for the scientific task (*p* = 0.0010 for patients and *p* = 0.0106 for healthy subjects). Further, the survey responses suggest that neither healthy subjects (*p* = 0.4652) nor patients (*p* = 0.0679) perceive one task to be more difficult than the other (Q4), and their responses fail to reach a significant difference (*p* = 0.0730). Consistent with task preference, results also support the expectation that, given a choice to repeat one of the tasks, both groups choose the scientific task (Q5). In fact, both healthy subjects (*p* = 0.0106) and patients (*p* = 0.0010) indicate that they would prefer to repeat task T over task NT, and such responses are not significantly different (*p* = 0.5517).

**Fig 4:**
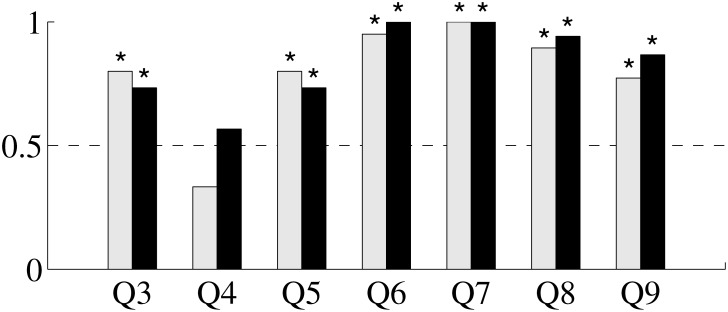
Bar plot of survey results for questions Q3–Q9 (see Table 1). Gray bars indicate the proportion of positive responses from patients, while black bars refer to healthy subjects (for Q6–Q9, neutral responses are omitted). An asterisk above the bar indicates a significant difference from chance. For Q6–Q9, the fraction of neutral responses is: 0.17, 0.57, 0.43, and 0.50 for healthy subjects, respectively, and 0.33, 0.63, 0.37, and 0.27 for patients.

**Table 1:** List of survey questions administered to all participants after the completion of both tasks.

Q3	which task did you like more?
Q4	which task was more difficult?
Q5	if you had to repeat one of the tasks, which would you choose?
Q6	circle the letter under the face that describes how identifying objects and animals made you feel.
Q7	circle the letter under the face that describes how moving the cursor without identifying animals and objects made you feel.
Q8	rank how easy or difficult identifying objects and animals is.
Q9	rank how easy only moving the cursor without identifying objects and animals is.

### Dissecting the elements contributing to individual preference (Fig. 4) Q6–Q9

Both patients (*p* < 0.0001) and healthy subjects (*p* < 0.0001) associate a positive feeling with tagging images (Q6). Both groups (*p* < 0.0001) consider moving the cursor to be positive (Q7), and the responses between the groups do not significantly differ (*p* = 0.6073). The responses of healthy subjects and patients regarding the difficulty of image classification (Q8) are indistinguishable (*p* = 0.9061), with both healthy subjects’ and patients’ (*p* < 0.0001) concurring on the assessment that such a task is not difficult. The two group’s responses are not statistically different when ranking the difficulty of moving the cursor (Q9) (*p* = 0.3570), with the responses of healthy subjects (*p* < 0.0001) and patients (*p* = 0.0019) indicating it to be not difficult.

### Individual performance in path following (Fig. 5)

Consistent for each task, the healthy group of participants score significantly better for normalized path length and mean arrest period ratio, in addition to path duration (*p* = 0.0076, *p* = 0.0033, and *p* = 0.0076 for normalized path length, mean arrest period ratio, and path duration in task T, respectively, and *p* = 0.0054, *p* = 0.0062, and *p* = 0.0444 in task NT).

**Fig 5:**
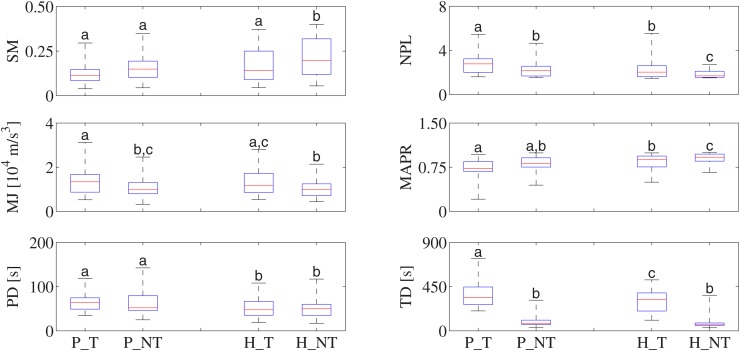
Speed metric (SM), normalized path length (NPL), mean of jerk (MJ), mean arrest period ratio (MAPR), path duration (PD), and task duration (TD) for both groups and both tasks, where P_T, P_NT, H_T, and H_NT denote respectively patients task T, patients task NT, healthy subjects task T, and healthy subjects task NT. Results are given as box plots, where the red line is the median, edges are the 25th and 75th percentiles, and whiskers extend to the extremes of the data. Whiskers not sharing a common letter are statistically different.

Between the two groups for each task, the mean of jerk is not significantly different (*p* = 0.4918 and *p* = 0.4965 for tasks T and NT, respectively). However the other indicator of movement smoothness, the speed metric, is significantly better in the healthy population for task NT (*p* = 0.0166), but not significantly different between the two groups for task T (*p* = 0.1580).

Some significant differences in performance metrics are found within groups and between tasks. Considering patients, the normalized path length for task T of 2.91 is significantly greater than that of 2.39 for task NT (*p* = 0.0014). Task NT for patients also has a significantly lower mean of jerk (*p* = 0.0098). Similarly, healthy subjects generate trajectories with significantly smaller normalized path length and mean of jerk for task NT (*p* = 0.0117 and *p* = 0.0002, respectively), indicating that both groups performed better on task NT. Additionally, healthy subjects have a significantly higher mean arrest period ratio for task NT (*p* = 0.0407).

Counting the time from the beginning to the end of each task (the task duration), Task T requires significantly more time (about four times as much) than task NT for both patients (*p* < 0.0001) and healthy subjects (*p* < 0.0001).

## Discussion

In this study, we show that including a web-based citizen-science participation module in a robotics-based low-cost rehabilitation platform is a viable means to engage individuals in therapeutic exercises. The platform consists of an off-the-shelf haptic joystick and laptop computer. Individuals use the joystick to participate in a citizen science project wherein the performance of scientific tasks is varied in two scenarios. The setup allows for administering repetitive exercises often characteristic of rehabilitation treatments [3], while also capturing sensor data sufficient for constructing a set of performance metrics that can be used for rehabilitation assessment [18, 52, 53].

### Including citizen science tasks increases participants’ engagement in rehabilitation tasks

An important finding of this study lies in the use of citizen-science in rehabilitation exercises. Specifically, our results show that including scientific tasks increases preference for rehabilitation. A key issue in rehabilitation regimens is the willingness of patients to perform the prescribed exercises [22], where motivation has been identified as a significant factor in following the prescribed regimen [25–27]. As such, emphasis has been placed on providing avenues for motivating patients. The use of video games is widely explored in this context, however motivation for gaming is shown to vary with both age [56] and gender [57]. Building on the work presented in [39], we have here presented an interactive rehabilitation system that acts on multiple motivational drivers. While [39] leverages science learning that targets intrinsic motivation [44], active science participation through citizen science considered herein addresses a wider spectrum of motivations. Indeed, by performing science tasks users are motivated both by personal interest and by contributing to science [45].

Interestingly, in choosing to repeat the citizen science task, participants are electing to perform the task that takes significantly more time. This is particularly telling when considering that increasing the time spent performing exercises may be beneficial to the rehabilitation process [58]. In this context, the work reported here shows that not only does the inclusion of citizen science tasks increase preference for exercises and willingness to repeat them, but it also motivates individuals to perform exercises that require more time than those without. While this study was based on a virtual environment unfamiliar to the participants, we expect motivation to further increase by providing virtual environments that are more relevant to the participants, since involvement in community-based online activities such as mapping has been found to originate primarily from the geographic region where the project takes place [59].

The result that the difficulty of the exercise as experienced by participants is similar across both conditions (with and without the citizen science task) and groups is also an important finding. The act of typing words using the joystick to tag the images requires precise positioning of the joystick to select each letter, which is expected to be perceived as a difficult task for motorically impaired participants, who generally have difficulties in using computer peripherals, such as a mouse and keyboard [60]. The excitement of contributing to science may compensate for this perceived difficulty, and thereby allow participants to enjoy performing additional tasks.

### The low-cost setup provides useful data for quantifying sensorimotor performance

Accurately quantifying an individual’s sensorimotor performance is an important factor in judging the severity of their impairment and tracking their progress in the recovery of motor function. For the proposed low-cost setup to be a feasible method for performing rehabilitation exercises in the absence of a physical therapist, the device must be able to collect sufficient data for detecting the different levels of performance of healthy subjects and patients.

Further interpreting the findings that healthy subjects in general score better on normalized path length, mean arrest period ratio, and path duration, we evince that not only do healthy subjects perform path following in a quicker time and with fewer pauses, but they also do it more accurately than patients. While an apparent increase in performance of the healthy group when compared to the group of patients may be ascribed to the age difference between the two groups, motor performance typically increases with age [61]. Given that the mean age of the patients is greater than that of the healthy subjects, we propose that deficiencies in patients’ performance with respect to the healthy subjects are likely due to their pathology, and not their age. The better performance on the speed metric for healthy subjects supports the expectation that healthy subjects have smoother movements than patients [18]. Similarly, a high mean of jerk in patients was expected due to less smooth movements [18], but the mean of jerk was found to not be significantly different between two groups. This may be explained by the significantly lower path duration. Given that the healthy subjects cover the same path in a shorter amount of time, it may be reasonable to expect higher levels of jerk. In other words, if the participants were told to take a specific amount of time to travel the path, a difference in the mean of the jerk of the trajectories between patients and healthy subjects may arise.

Both patients and healthy subjects score better on normalized path length and mean of jerk in task NT. The decrease in path-following performance associated with task T for both groups may be due to the shift in attention required in task T from following a path to tagging an image, and back to following a path, while in task NT the participant is focused only on following the path for the duration of the trial. Furthermore, task T has a longer break between path following segments due to the time required for tagging. Such a break may negatively impact the participants’ retention of their learned path following ability, thereby degrading performance [62]. Additionally, while in task NT the primary (and sole) objective is to follow the path, and the additional objective in task T of tagging images may take precedence and be more engaging in the eyes of the participant. In other words, in task T, the participant may sacrifice the accuracy of path following to reach the additional goal of tagging an image, where accuracy of movements has been shown to be affected by focus [63]. The finding that both groups score better on speed metric in task NT is consistent with the results reported in [39], where healthy subjects exhibited a significantly better speed metric in a path following task in the absence of science learning.

### Citizen science projects and rehabilitation may benefit from each other

The present study demonstrates that an interactive system, which provides a context for images to be tagged within a game-like environment, is enjoyable for both healthy subjects and patients, who take part in an authentic research project. Participants indeed contribute to Brooklyn Atlantis by gathering useful data in the form of image tags. In this specific empirical study, the participants’ contribution to citizen science is limited to the identification of canoes, birds, and trees in a predefined set of six images. In future studies, we will enable web-based capabilities of the platform, to engage each participant in the analysis of a wider variety of images, thereby increasing the overall citizen science contribution.

While rehabilitation has pursued videogame integration with the aim of increasing compliance [64–66], citizen science has seldom also leveraged game-like components to increase participation [67]. In *Foldit*, for example, citizen scientists compete and collaborate in a game-like environment for identifying protein structures [68], and the *ESP game* utilizes a gaming environment to motivate a pair of users to label images online [69]. Targeting additional motivation drivers related to gaming, such as exploring a map through a haptic device as we propose here, may prove successful in enhancing participation in citizen science projects, which are often limited to a relatively small number of active volunteers [70].

Without background information on the project, the act of tagging a series of images may be perceived as extraneous work. Thus, providing participants with context for their task, such as navigating a virtual environment, offers an added degree of meaning for their contribution, which may be important for fostering commitment [71]. Future work will further elucidate this hypothesis by considering additional experimental conditions, in which image tagging is not associated with a citizen science project.

### Conclusions

In conclusion, this study indicates that citizen science is an effective means to engage patients in rehabilitation exercises. The system utilized in this study, consisting of a low-cost haptic joystick and laptop computer, is also able to sufficiently quantify performance, enabling its use in a rehabilitation setting. The results show that groups of healthy subjects and patients undergoing rehabilitation treatments prefer performing an exercise with a citizen science component, and would choose to repeat it. The data gathered from the low-cost haptic device permits an individual’s trajectory to be quantified in terms of a set of performance metrics. Comparing the performance metrics of patients with those of healthy subjects, significant differences in the trajectories are observed, where healthy subjects generally score better. These findings suggest that this low-cost system can effectively provide useful data for evaluating a patient’s level of recovery, where the performance metrics of patients are expected to approach those of healthy subjects as they recover.

This study involved only children as participants. We expect some of the findings to vary with increasing age, as age-related differences have been demonstrated in videogame play, for example, where adolescents were found to play videogames less often as age increases, but for longer time periods [72]. In future studies, the role of age on engagement in the proposed exercises, as well as means for tailoring the system to various age groups, will be investigated.

Ultimately, we anticipate this system to find application as a low-cost platform for telerehabilitation [73] for at-home treatments following hospital discharge. As patients perform their prescribed exercises while contributing to a science project, the trajectory data from each trial can be automatically sent to a physical therapist, who may also remotely set the level of intensity of the force feedback.

## Supporting Information

S1 Dataset Raw trajectory data collected by the haptic device, set 1. (ZIP)

S2 Dataset Raw trajectory data collected by the haptic device, set 2. (ZIP)

S3 Dataset Raw trajectory data collected by the haptic device, set 3. (ZIP)

S4 Dataset Raw trajectory data collected by the haptic device, set 4. (ZIP)

S5 Dataset Raw trajectory data collected by the haptic device, set 5. (ZIP)

S6 Dataset Raw trajectory data collected by the haptic device, set 6. (ZIP)

S1 Table Participant survey responses. (XLS)
